# Corrosion Monitoring Method of Porous Aluminum Alloy Plate Hole Edges Based on Piezoelectric Sensors

**DOI:** 10.3390/s19051106

**Published:** 2019-03-05

**Authors:** Wei Dai, Xiangyu Wang, Meng Zhang, Weifang Zhang, Rongqiao Wang

**Affiliations:** 1 School of Reliability and Systems Engineering, Beihang University, 37 Xueyuan Rd., Haidian Dist., Beijing 100191, China; dw@buaa.edu.cn (W.D.); zhangmeng123@buaa.edu.cn (M.Z.); 2 School of Energy and Power Engineering, Beihang University, 37 Xueyuan Rd., Haidian Dist., Beijing 100191, China; wangxiangyu2016@buaa.edu.cn (X.W.); wangrq@buaa.edu.cn (R.W.); 3 Collaborative Innovation Centre for Advanced Aero-Engine, Beihang University, 37 Xueyuan Rd., Haidian Dist., Beijing 100191, China

**Keywords:** corrosion damage detection, reconstruction algorithm, Lamb wave, porous aluminum alloy plate

## Abstract

Corrosion damage to the aircraft structure can significantly reduce the safety performance and endanger flight safety. Especially when the corrosion occurs in a stress concentration region, such as hole edges, it can easily threaten the entire structure. In this paper, an on-line imaging qualitative monitoring algorithm based on piezoelectric sensors is proposed for detecting hole edge corrosion damage of porous aluminum alloy structures. The normalized amplitude is used to characterize the correlation between the initial Lamb wave signal and the damage signal, which is as an image reconstruction parameter in the algebraic iterative probability reconstruction algorithm. Moreover, a homogenization algorithm is proposed to process the reconstruction results. The experimental results of single hole and double hole corrosion for porous aluminum alloy plate show that the method can effectively achieve the location and quantification of corrosion damage to one and two holes of the porous structure.

## 1. Introduction

Aluminum alloys are widely used in aircraft manufacturing due to their high strength and low density [1]. As the aircraft’s service time increases, the aircraft structure is exposed to environmental corrosion with increasing proneness. One of the main causes of aluminum alloy structural failure is corrosion damage. According to the US Federal Aviation Administration, corrosion damage directly or indirectly caused more than 30% of annual maintenance and repair costs for US Navy and Air Force aircraft [2,3]. Corrosion damage is concealed, extensive and random [4,5]. Bolted joints are widely used in aircraft structures, not only due to the ease of fabrication and the flexibility provided by bolted joint structures, but also due to the higher resilience against shocks, greater damping against external excitations, and enhanced endurance. When the hole edge is corroded, the stress concentration is intensified, resulting in a unsafe state or even failure of the structure [6,7,8]. The previous methods for identifying aircraft corrosion damage involve expensive, labor intensive periodic inspections, which lead to the reduction of aircraft availability.

The sensors can detect the structural corrosion timely and avoid unnecessary losses. As the technology is applied to sensors in different environments, structural locations and metal matrix with different properties, different types of corrosion sensors have been studied [9,10,11,12,13,14]. There are many kinds of corrosion sensing technology. According to whether the corrosion results can be obtained directly, it can be divided into direct monitoring and indirect monitoring. Corrosion monitoring which can directly obtain a corrosion result (such as corrosion weight loss, corrosion current, etc.) is called direct monitoring, otherwise it is indirect monitoring. Direct monitoring techniques are only suitable for large area monitoring. Direct monitoring technology includes linear polarization based monitoring, resistance probe technology, electrochemical noise analysis method, etc. For the indirect monitoring technology, since the piezoelectric sensor is light, it can monitor the spread of corrosion damage in real time remotely, and multiple piezoelectric sensors can be used to locate the corrosion damage location. Indirect monitoring technology includes optical sensor technology, acoustic emission technology, PH value method, piezoelectric components and corrosion indicator paint, etc. Some corrosion damage monitoring methods based on piezoelectric sensors have been described. Visalakshi et al. [15,16] verified that PZT sensors were useful for monitoring corrosion damage on a bare rebar via experiments. Rathod et al. [8] used a symmetry breaking pattern algorithm to identify the location of plate-like aluminum corrosion damage. Wang et al. [17] proposed a sparse representation strategy based on an 11-norm optimization algorithm for guided-Lamb-wave-based inspections. Thomas et al. [7] applied circular array of piezoelectric wafer active sensor to detect material loss in thin plates. Yu et al. [2] used PWAS for the corrosion damage in plate-like aluminum structure. Recently, the detection of circle-like corrosion defects in thin plates was also investigated, Sedaghati et al. [18] proposed a technique of Lamb wave omnidirectional generation, which was later testified by himself. Rao et al. [19] achieved accurate thickness reconstruction of corrosion damage by using the dispersive regimes of selected guided wave and a reconstruction algorithm. In early 20th century, Kaczmarz and Cimmino proposed algebraic iteration methods for solving linear systems [20]. In 1970, Gordon, Bender and Herman applied the Kaczmarz method to the medical field [21], which was called the Algebraic Reconstruction Technology (ART). Chen et al. [22] developed a Lamb-wave-based identification approach for evaluating damage in particular in submerged structures. B. Jiang et al. [23] used a CT method for damage for damage inspections of a steel tube slab (STS) structure [24,25,26,27,28,29,30]. However, the damage in the framework of integrated structural health monitoring, such as hole-edge corrosion and multi-hole edge corrosion, is rarely reported in published literature [31,32].

A hole edge corrosion monitoring experiment for porous aluminum alloy structures based on Lamb waves is designed in this paper. This experiment simulates a real rivet hole corrosion environment, and the imaging method of ART algorithm is used. The original tomographic imaging matrix results in sparse matrix, which cannot accurately represent the damage area. So a homogenization algorithm is proposed to qualify the damage. This method can guide the engineering applications in monitoring the hole edge corrosion of porous aluminum alloy structures.

In this study, a corrosion monitoring method has been developed in a porous aluminum alloy plate. A(0) mode of Lamb wave is employed to detect the damage based on computerized tomography technique and ART is used to pursue the CT image. Homogenous algorithm is used to quantify corrosion damage. In the forthcoming section of this article, a brief description of guided wave, selection of characteristic parameters and imaging algorithm are presented to be followed by hole edge corrosion monitoring of single hole and double hole for porous aluminum alloy plates, and finally the quantitative analysis of the imaging results are carried out.

## 2. Background

### 2.1. Guided Wave in Aluminum Alloy

**Figure 1:**
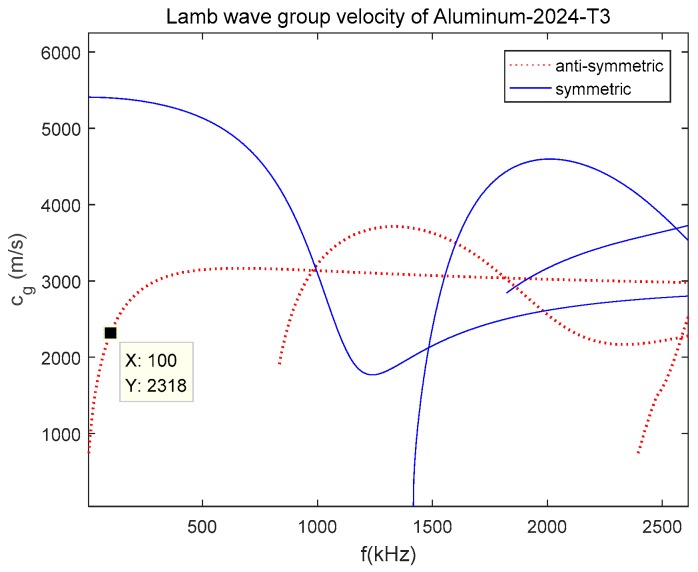
Dispersion curve for a smooth aluminum alloy plate with thickness of 2 mm.

Porous aluminum alloy plate has a wide area of applications. In fact, the long-term durability and reliability of porous aluminum alloy are not fully understood. For this reason, the development of methods to perform reliably in situ SHM of porous aluminum alloy has received extensive attention. For smooth plate structures with isotropic material such as the aluminum alloy, the guided waves are a result of the longitudinal P-waves and transverse S-waves fulfilling the traction-free boundary condition. The dispersion curve for a plate with thickness of 2 mm made out of aluminum alloy material (Young’s modulus of 72.4 GPa, Poisson’s ratio of 0.33, density is 2780 kg/m^3^) is plotted as group velocity against frequency (Figure 1), where anti-symmetric mode A(m) and symmetric mode S(m) represent two modes of guided waves in aluminum alloy plate, and m represents the sequential order of mode. Lamb wave modes are dispersive waves and their velocities are function of the frequency-thickness production. The group velocity dispersion curve shows that wave motions in aluminum alloy plate are dispersive, and there exists multiple modes for a particular frequency [33,34]. To eliminate the dispersion and to minimize the number of modes, a narrowband signal with frequency centered around 100 kHz is selected. At this frequency, there are two possible modes, the anti-symmetric mode A(0) and symmetric mode S(0). The A(0) mode was employed for corrosion identification in this study. The reasons are as follow, (i) For the interaction between wave and damage, the half wavelength of the selected mode must be shorter than or equal to the damage size. A(0) mode has shorter wavelength and higher sensitivity to small size damage such as corrosion damage, under given excitation frequency; (ii) Compared with S(0) mode, A(0) mode has lager signal magnitude and higher signal-to-noise ratio at relatively low frequencies such as 100 kHz used in this study [22,35,36,37].

### 2.2. Selection of Damage Characteristic Parameters

The corrosion produces wave diffraction and mode conversion that can be analyzed and compared with the pristine signals. Analysis of the change in the guided wave shape, phase [38], and normalized amplitude should yield in dictations about the corrosion presence.

Generally, when signal processing is performed, the normalized amplitude of the signal, the phase of the signal, and the correlation coefficient of the difference signal are extracted for subsequent image processing. 

**Figure 2:**
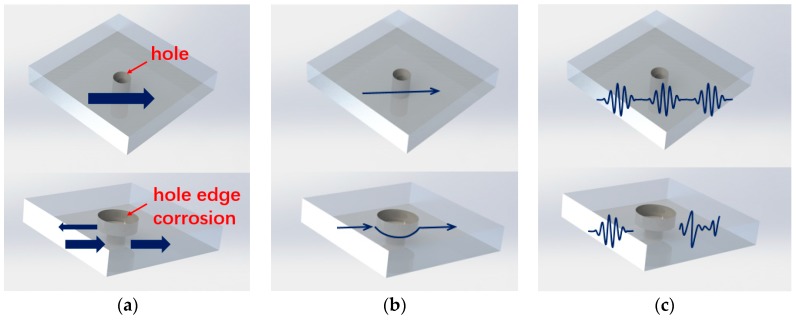
Illustration of changes for Lamb waves passing through a hole-edge corrosion. (**a**) Signal maximum amplitudes, (**b**) Signal phase, and (**c**) Signal time series.

Mechanisms for choosing these three features are illustrated in Figure 2. When Lamb waves pass through a region with discontinues, because of forward scattering, the transmitted waves are modified. As shown in Figure 2a, the normalized amplitude of the signal reflects the energy of the signal, because the upper part of the plate is discontinuous due to corrosion damage, the signal will be reflected and scattered at this location. Thus, the normalized amplitude of signal can serve as a damage characteristic feature for corrosion size. The performance of piezoelectric sensors used to excite and receive Lamb wave signals on each sensing path proves to be discrete. In order to reduce the error caused by the sensor, the amplitude is normalized by the amplitude of the health signal to obtain a normalized amplitude. The normalized feature selected based on damage diagnosis feature parameter normalization method is only related to the relative difference between the damage signal and the reference signal, and is independent of the absolute amplitude of the signal, the arrangement of the sensor, the signal propagation distance. The structure and material properties are irrelevant. Determining the threshold for the presence of structural damage on this basis can form a standard for measuring damage generation and expansion. When the Lamb wave passes through the corrosion, it expands around the edge of the corrosion as shown in Figure 2b, increasing the propagation time and characterizing the phase delay in the time domain signal. Corrosion damage is a thickness-variable damage. This type of damage causes a change in the frequency-thickness product to change the group velocity and phase velocity of the received signal, thereby changing the phase angle of the signal. Another feature is correlation coefficient. The correlation coefficient is a statistical concept that characterizes the similarities and differences between the current state of the signal and is a ‘pristine’ situation as shown in Figure 2c. As the result of the damage in the excitation sensing path affects the signal, the ratio of the Lamb wave signal to the health signal will change. As the size of the damage increases, the value of the correlation coefficient will gradually decrease relative to the initial state, and the amount of change also indicates the size of the corrosion damage area. The mathematical definition of the correlation coefficient is ρ, which is based on two sets of data, $s_{j},\;s_{k}$:

(1) $$\rho =\frac{\mathrm{Cov}\left(s_{j},s_{k}\right)}{\sigma_{s_{j}}\sigma_{s_{k}}}$$

where $\mathrm{Cov}\left(s_{j},s_{k}\right)$ is the covariance of $s_{j}$ and $s_{k}$, $\sigma_{s_{j}}$ and $\sigma_{s_{k}}$ are the variances of $s_{j}$ and $s_{k}$, respectively. In the formula, the covariance Cov expression is:

(2) $$\mathrm{Cov}\left(s_{j},s_{k}\right)=\frac{\sum_{i}^{E}\left(s_{j}\left(t_{i}\right)-{\bar{s}}_{j}\right)\left(s_{k}\left(t_{i}\right)-{\bar{s}}_{k}\right)}{E}$$

where E is the number of data sets, that is, the number of sensor monitoring paths. The standard deviations in the formula $\sigma_{s_{j}}$ and $\sigma_{s_{k}}$ is:

(3) $$\sigma_{s_{j}}=\sqrt{\overset{E}{\underset{i}{\sum }}{\left(s_{j}\left(t_{i}\right)-{\bar{s}}_{j}\right)}^{2}}$$

(4) $$\sigma_{s_{k}}=\sqrt{\overset{E}{\underset{i}{\sum }}{\left(s_{k}\left(t_{i}\right)-{\bar{s}}_{k}\right)}^{2}},$$

Among them, a set of data represents an A(0) wave packet obtained by a pair of excitation sensing paths. The health signals $\left(s_{j}\right)$ are acquired after the test equipment is built, then the Lamb wave signal group with corrosion damage are collected. Corrosion damage is quantified by using a tomography algorithm based on the correlation coefficient as the damage characteristic parameter.

When monitoring the hole-edge corrosion of a single-hole structure, correlation coefficient was selected as characteristic parameter. When monitoring the hole-edge corrosion of the porous structure, the normalized amplitude is selected. This happens because the dispersive behaviour of a porous aluminium allay plate is different from a single aluminium allay plate. For corrosion damage on the smooth plate, when the Lamb wave passes through the corroded area, as the thickness of the plate becomes thinner, the frequency thickness product changes, and the group velocity of the wave which is obtained by the dispersion curve becomes slower, so that the phase of the wave changes. There is reflection in the corroded area wave, which changes the normalized amplitude. Therefore, the correlation coefficient can be extracted as the damage feature value to obtain a good damage prediction result.

For corrosion damage on the porous plate, when the signal passes through the corrosion region of the hole edge, the dispersion of the wave are more obvious due to the existence of the hole, and the normalized amplitude changes significantly. The reflection of the signal is more complicated due to the existence of the hole. Since the corrosion damage occurs at the edge of the hole, the phase velocity change caused by the change of the frequency and thickness caused by the corrosion region, the contribution of the corrosion region on the change of the signal is relatively small compared with the reflection superposition of the Lamb wave generated by the hole. Therefore, corrosion damage cannot be effectively monitored. Therefore, when the phase angle change is used as the feature amount of the edge corrosion damage monitoring, the imaging result is not ideal. However, the correlation coefficient can be extracted as the damage feature value to obtain accurate damage prediction results. It is proved that the correlation coefficient has not changed much, and the normalized amplitude has changed a lot. For the corrosion damage on the multi-hole plate, the number of holes is significantly increased compared with the single-hole plate, making the Lamb wave superposition more obvious. This process is equivalent to adding amounts of boundary conditions in the Lamb wave propagation path, so that the dispersion phenomenon of the Lamb wave gets more obvious. This is to say, the phase difference cannot reflect the damage characteristics. Therefore, the normalized amplitude is selected as the damage feature value.

### 2.3. Proposed Data Processing Method

For the acquired signals, the ART algorithm is used. When the ART algorithm is applied, the monitoring area of the entire aluminum alloy plate is discretized. The reconstructed image h(x,y) is composed of N=n×n square non-overlapping pixel grids. Each side of the square has a side length of 1, and the pixel value of the *j*-th pixel grid is represented by $h_{j}\left(1\leq j\leq N\right)$.

**Figure 3:**
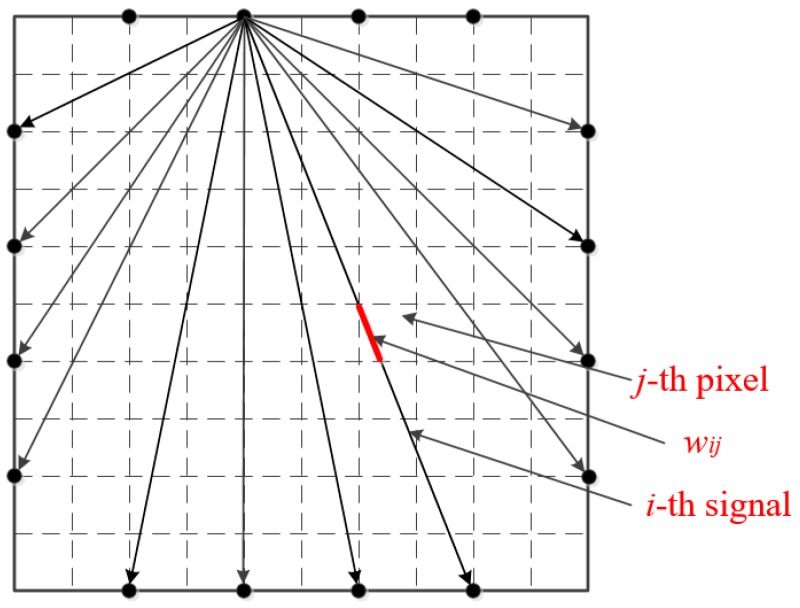
ART algorithm application diagram.

As shown in Figure 3, there are a total of *M* signals that are to be projected and scanned for the reconstructed image. $p_{i}$ is used to represent the projection value of the *i*-th (1 ≤ *i* ≤ *M*) signal, that is, the line integral value of the ray along the ray path on the image to be built. The length of the *i*-th signal scanned on the *j*-th pixel grid, that is, the weight factor, is represented by $w_{ij}$.

The relationship between them is as follows:

(5) $$\overset{N}{\underset{j=1}{\sum }}w_{ij}h_{j}=p_{i},\;i=1,2,\cdots ,M$$

which is:

(6) wh=p

Among them:

(7) $$h={\left(h_{1},h_{2},\cdots ,h_{j}\right)}^{T}$$

(8) $$w=\left(w_{i1},w_{i2},\cdots ,w_{iN}\right),i=1,2,\cdots ,M,$$

(9) $$p={\left(p_{1},p_{2},\cdots ,p_{1}\right)}^{T},$$

**Figure 4:**
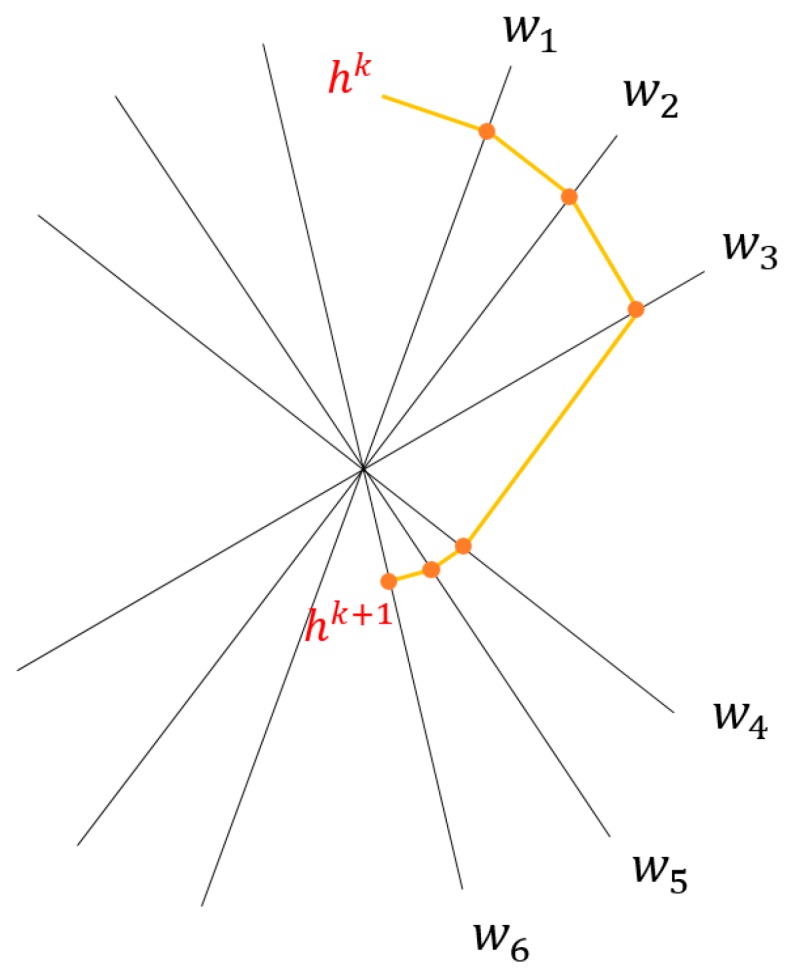
One iteration with the relaxation parameter $\lambda_{k}=1$.

Equation (6) has *N* unknowns and *M* equations. In practical calculations, *M* = 96 and *N* = 6400. Because the *M* and *N* values are very large, it is almost impossible to solve by using a general solution of the linear equations. Thus using the following iteration method, an initial value is given first when iterating, then calculated according to Equation (10):

(10) $$h_{i}^{k}=h_{i-1}^{k}-\lambda_{k}\frac{h_{i-1}^{k}\cdot w_{i}-p_{i}}{w_{i}\cdot w_{i}}w_{i}$$

where *k* is the number of iterations, $h_{i}^{k}$ expresses the value of the vector *h* when the *i*-th equation participates in the calculation at the *k*-th iteration and $\lambda_{k}$ is the relaxation factor, which the general value is 0~2. Figure 4 shows an example of one iteration for the consistent case with the relaxation parameter $\lambda_{k}=1$.

After all *M* equations have been used, one iteration is completed, and the value of *h* obtained at this time is taken as the initial value of the next iteration.

### 2.4. Proposed Homogenization Processing Method

**Figure 5:**
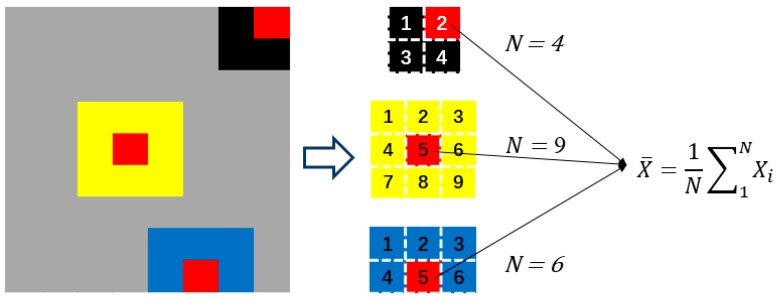
The method of homogenization.

**Figure 6:**
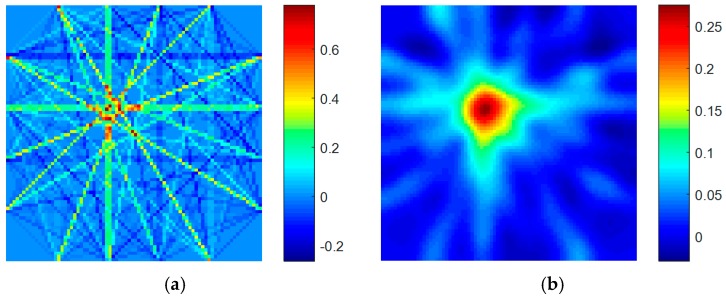
The imaging results (**a**) without homogenization and (**b**) with homogenization.

After getting the initial matrix, we need to process the matrix to get the detail image. Since each specific sensor path is affected by its path, the final result is obtained. To better simulate this process in final reconstructed discretization sampling results, a homogeneous simulation method was used to construct new images using the original images. The dimension of the new image is the same as the original image, but the value in each grid is the average of the values of all adjacent grids of the grid corresponding to the corresponding positions in the original image. When the pixel grid is located at the four corners of the monitoring area, the pixel value of the grid is an average of four grid pixels; when the pixel grid is located at the edge of the monitoring area, the pixel value of the grid is an average of the adjacent six grids; when the pixel grid is located at the other positions, the pixel value of the grid is the average of the surrounding nine grids. The schematic diagram is shown in Figure 5. The results of homogenization method can be seen in Figure 6.

## 3. Corrosion Tests

### 3.1. Experiment Equipment

The instrument system used in this experiment is the SHM-ISS-4.0A integrated structural health monitoring scanning system produced by Nanjing Kingsmart Monitoring Technology Co., Ltd. (Naijing, China). The overall monitoring system can achieve a large area, multi-site active health monitoring scans by designing a large-scale piezoelectric sensor array. Its main technical indicators are shown in Table 1. 

**Figure 7:**
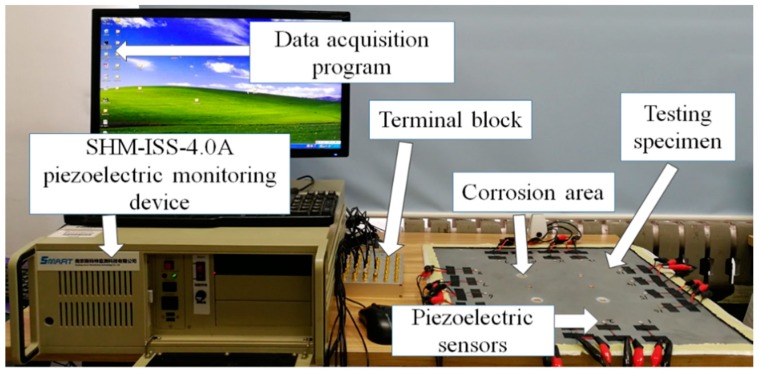
The experimental device.

The overall test setup is shown in Figure 7, which includes a SHM-ISS-4.0A piezoelectric monitoring device, piezoelectric sensor, digital acquisition software, terminal board, and testing specimen. The PZTs were connected with thin insulated wires to a 32-channel terminal block. The SHM-ISS-4.0A piezoelectric monitoring device was used to generate a 5-V smoothed 50 to 200-kHz tone burst excitation and it also can collect the signals captured at all PZTs and a data acquisition program was used. 

### 3.2. Test Plan Design

Traditional guided wave SHM techniques developed from non-destructive evaluation (NDE), such as pitch-catch method, pulse-echo method, etc., depend on the availability of a pristine structure baseline to assess the structural health. In the pulse-echo method, flaw are detected in the form of additional echoes. The pitch-catch method can be used to detect structural changes that take place between a transmitter and a receiver transducer. The aim of embedded pitch-catch NDE is to detect corrosion in aluminium alloy. The two classic layout of the piezoelectric sensor are the pulse-echo configuration and pitch-catch configuration. The pitch-catch configuration was selected because it is easier to implement a complete scan for each path.

**Figure 8:**
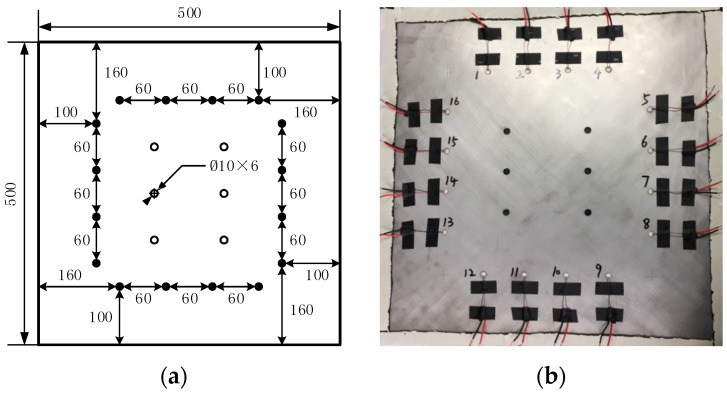
(**a**) The diagram of the layout of the piezoelectric sensor network and (**b**) the real layout of piezoelectric sensor network (unit: mm).

**Figure 9:**
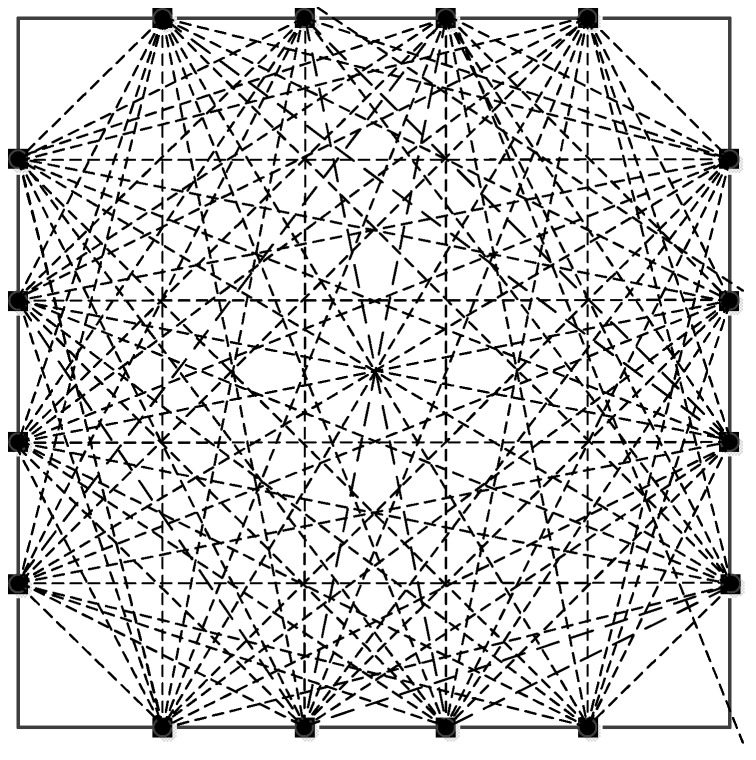
The layout of piezoelectric sensor network.

The diagram of the layout of the piezoelectric sensors on the tested structure is shown in Figure 8, the thickness is 2 mm. 16 piezoelectric sensors are arranged on the structure, and 96 effective sensing paths was achieved (shown in Figure 9). The related studies have shown that the layout method is more effective for thickness variation damage.

### 3.3. Corrosion Solution

The acid solution was used to simulate the hole edge corrosion test on the sample. The hydrofluoric acid solution was provided by Hengxing Reagents (Tianjin, China). The chemical composition is shown in Table 2.

The two holes on the test structure were corroded by using 15 mL of 8% hydrofluoric acid solution and kept it for 11 h at 23 °C. 15 mL of an 8% hydrofluoric acid (HF) solution was used to obtain the hole-edge corrosion of the 2024 aluminium alloy plate required for the experiment. First, the corrosion-resistant PVC pipe with an inner diameter of 18.66 mm is fixed at the hole edge corrosion position, and an etching solution groove is formed on the surface of the aluminium plate. In order to prevent the corrosion solution from flowing out of the preset hole, the test piece was fixed with 401 glue on the back side of the corrosion hole. After the 401 glue is solidified, 15 mL of hydrofluoric acid is taken up using a medical syringe and completely injected into the PVC tube corrosive solution tank. In the preliminary test, it is found that hydrofluoric acid corrodes the surface of the aluminium plate, and bubbles are generated. When no bubbles continue to be produced in the tank, it indicates that the corrosion has been completed. Then remove the PVC tube, 401 glue and plastic sheet to collect the signal.

### 3.4. Excitation Frequency Design

In the single-hole edge corrosion test, it has been found that 100 kHz is more sensitive to the edge corrosion damage of a single-hole structure. However, when the Lamb wave propagates in the porous aluminium alloy plate, the boundary reflection is serious and the Lamb wave has dispersion characteristics. The received signal is more complicated than that in a single-hole structure. Therefore, the centre frequency of 50 kHz to 200 kHz is selected to find out the suitable centre frequency to monitor porous aluminium alloys plate. 

**Figure 10:**
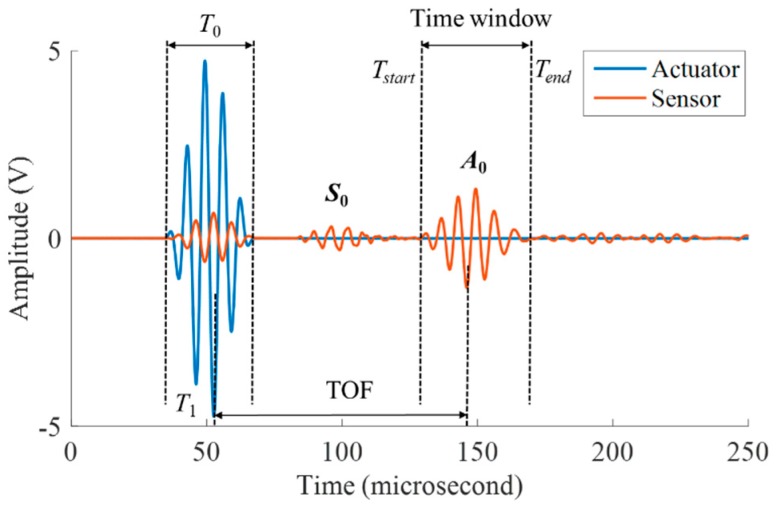
Schematic illustration for ToF and the time window calculation.

Time of flight (ToF), defined as the time consumed for a wavepacket to travel a certain distance, is one of the most straightforward features of a wave signal [39]. The diagram of ToF in this article is shown in Figure 10. Choosing an odd frequency is good for finding the peak value of the signal and making it easier to find the ToF (time of flight), so it is usually 3 or 5 cycles. For a tone-burst excitation with certain count number $N_{B}$, the higher the frequency, the shorter the time duration, and hence the wider the spectral spreading. To maintain the concentrate of the tone burst energy in a tight frequency band, when the frequency is reach 200-kHz, 5-cycle tone burst count was chosen instead of 3-cycle.

Tone burst signals were sent from one of the piezoelectric discs and collected at all the other piezoelectric discs, in a round-robin fashion. The tone burst frequency was swept over the 50- to 200-kHz range. A(0) Lamb waves travelling in the plate were scattered by the damage.

The excitation signal in our study consisted of a smoothed 5-count sine tone burst. The five cycles tone burst was obtained from a pure tone burst of these frequency filtered through a Hanning window. Figure 10 is a diagram of the time window. The Hanning window smoothing was applied to reduce the excitation of side frequencies associated with the sharp transition at the start and the end of a conventional (raw) tone burst. Through these means, it was intended that the dispersion effects would be minimized and the characteristics of wave propagation would be readily understood. The smoothed tone burst that resulted from this process was numerically synthesized and stored in PC memory as the excitation signal. The excitation voltage is 5 V, the sampling rate is 10 MSPS, and the sampling length is 5000 points.

The test firstly collected the array response signal as a reference signal implying the healthy structure, and then tested the corrosion damage on the first row and the first row of the sample in the first row of the sample by collecting the array signal through the SHM-ISS-4.0A device. The second hole was afterwards corroded by the same process and signals are also collected. Each set of data is collected three times and averaged to eliminate human errors and external environmental errors.

## 4. Results and Discussion

### 4.1. Case 1

**Figure 11:**
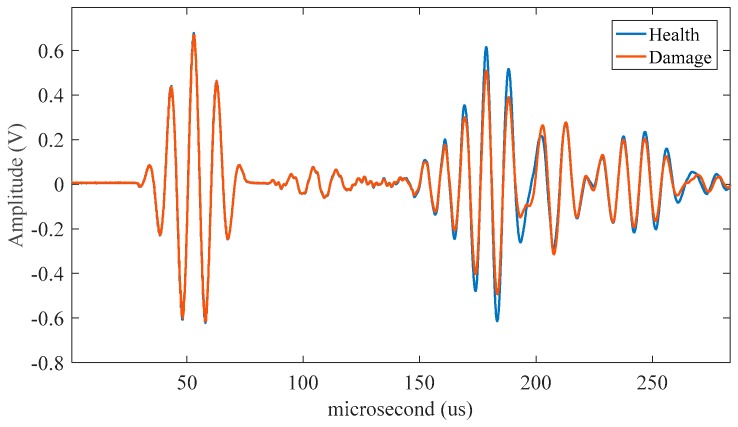
The Lamb wave signals without damage and with damage.

Health and damage signals were collected by SMART PZT device before and after corrosion. Each group of signals are collected three times to get the average value to eliminate the error caused by human error and the external environment in the test process. Figure 11 is the health signal and damage signal of the sensor path passing through the hole-edge corrosion damage. After collecting 96 paths of health signals and corrosive damage signals, the normalized amplitude is extracted as the characteristic parameter according to Section 2.2 analysis. ART algorithm was used to image, and then homogenization processing is carried out to obtain the final damage image.

**Figure 12:**
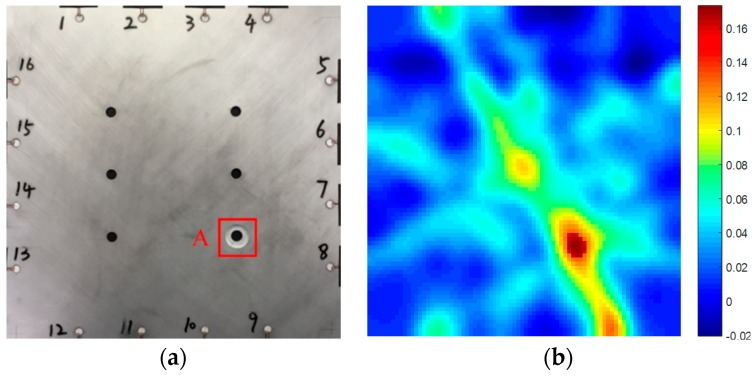
(**a**) The area of real one hole corrosion damage and (**b**) the area of predicted one hole corrosion damage.

As can be seen from the Figure 12a,b, the pixel value in the image is closely consistent with the position of the etching hole. In order to demonstrate the effectiveness of the method for monitoring corrosion damage of porous aluminium alloy structures proposed in this paper, the experimental prediction results are compared with the actual corrosion damage results. For the quantification of its area, the percentage error refers to the error between the predicted damage and the real damage. The percentage error δx was calculated by applying the following equation:

(11) $$\delta x=\frac{\left|x_{0}-x\right|}{x}\times 100\%$$

where *x* is the area of real corrosion and $x_{0}$ is the area of predicted one hole corrosion damage. After the experiment is completed, the specimen is cut and the real corrosion damage area is measured by an optical microscope.

**Figure 13:**
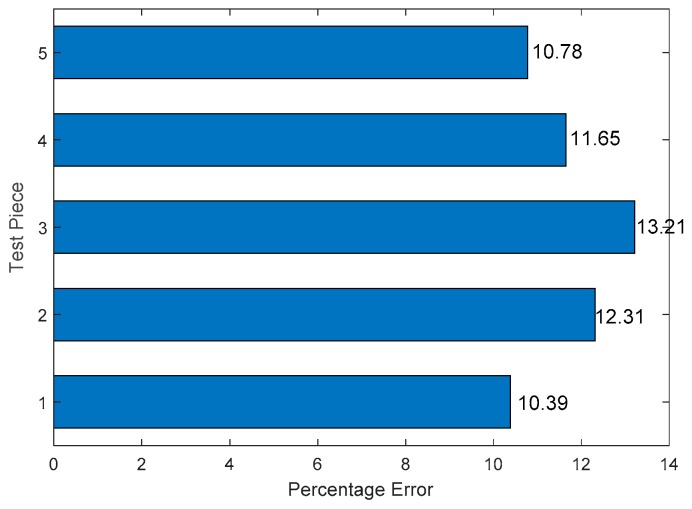
Percentage error of five test pieces.

In order to obtain enough data and reduce the uncertainty caused by the test error, five plates were designed as parallel specimens in this test as shown in Figure 13. The percentage error does not exceed 15% in one hole edge corrosion damage monitoring. In the one hole experiment, the normalized amplitude is used as the damage characteristic.

Figure 12 shows the real and predicted corrosion damage of a single hole. As shown in Table 3, in Figure 12b, when the pixel value is greater than 0.15, it is identified as a corrosion damage area. For the calculation, the predicted corrosion damage area of single hole is 301.44 ${\mathrm{mm}}^{2}$. The real corrosion damage area of single hole is 273.06 ${\mathrm{mm}}^{2}$. The percentage error between the predicted corrosion damage area and the real corrosion damage area is 10.39%. 

### 4.2. Case 2

After the one hole edge corrosion, a porous corrosion imaging exploration test was carried out, corrosion imaging was performed after the hole-edge etching of the hole B of the same plate in the same manner as in case 1. This experiment increases the signal interference between porous corrosion than case 1, making imaging quantification more difficult. 

**Figure 14:**
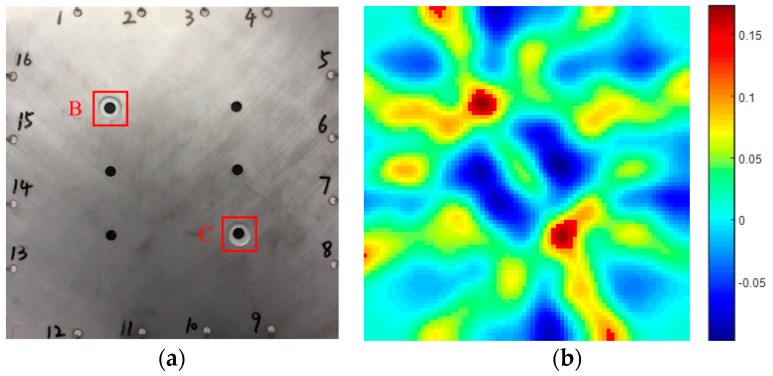
(**a**) The area of real two-hole corrosion and (**b**) the area of predicted two-hole corrosion.

Figure 14 shows the real and predicted corrosion damage of two holes. As shown in Table 4, in Figure 14b, where the pixel value is greater than 0.15, it is identified as a corrosion damage area. For the calculation, the single hole corrosion damage area predicted by the corrosion holes in the upper left corner is 329.31 ${\mathrm{mm}}^{2}$. The true single hole corrosion damage area is 273.06 ${\mathrm{mm}}^{2}$. The percentage error between the predicted single hole corrosion area and the true single hole corrosion damage area is 20.60%. The single hole corrosion damage area predicted by the corrosion hole in the lower right corner is 357.44 ${\mathrm{mm}}^{2}$. The true single hole corrosion damage area is 273.06 ${\mathrm{mm}}^{2}$. The percentage error between the predicted single hole corrosion area and the true single hole corrosion damage area is 25.75%. Thus for double-hole-edge corrosion monitoring under the same conditions, the considerable light leakage was observed owing to the excessive edge reflection caused by the existence of another hole. However, in spite of the light leakage, the position and size of corrosion spots were still able to be predicted. The results indicated that the Lamb-wave-based tomographic method can be used to monitor the multi-hole corrosion damage accurately.

The experimental results confirm that the proposed corrosion damage quantification method can accurately quantify the multi-corrosion damage of porous aluminium alloy structures.

## 5. Conclusion

A PZT-based active sensing method with improved sensor design has been used to detect the porous aluminium alloy plate hole-edge corrosion. The method uses lamb waves particularly first mode of anti-symmetric mode A(0), to detect the corrosion damage in plate. Two damage tomography tests for hole-edge corrosion have been conducted on one porous aluminium alloy plate specimen, with one having one hole-edge corrosion and the other having two hole-edge corrosion. ART algorithm is applied to provide the pixel matrix value. Homogenization processing method is used to process the sparse matrix to get the detail image. The corrosion area size is used to estimate the amount of corrosion size. Percentage error is used to detect the comparative difference between monitoring and actual result. Correlation coefficient is selected as the characteristic parameter in monitoring the corrosion of single hole structure, and normalized amplitude selected as the characteristic parameter in monitoring the corrosion of porous aluminium alloy plate. Through the above research, we can conclude that the on-line monitoring of aluminium alloy structure imaging qualitative algorithm based on piezoelectric sensor for hole edge corrosion damage of porous aluminium alloy structures can achieve the qualification the corrosion damage. 

## Figures and Tables

**Table 1:** Piezoelectric monitoring equipment technical indicators.

Device Number	SHM-ISS-4.0A
Maximum Length of Excitation Signal	40,000 data points
Vertical Accuracy	14-bit
Maximum Output Voltage	±50 V
Working Bandwidth	10 kHz–500 kHz
Sampling Rate	1 MHz–40 MHz
Sampling Accuracy	12-bit
Signal Input Range	±5 V

**Table 2:** The chemical composition of hydrofluoric acid.

**Molecular Formula**	HF	
**Content of HF**	≥40%	
**Impurity Content (%)**	Fe	≤0.0001
	Cl	≤0.001
	PO_4_	≤0.0002
	Heavy metal (Pb)	≤0.0005
	Fluorosilicate (SiF_6_)	≤0.04
	Others	≤0.004

**Table 3:** Damage simulation and prediction results.

	Damage Threshold	Actual Corrosion Area (unit is mm^2^)	Predicted Corrosion Area (unit is mm^2^)	Percentage Error
Hole A	0.15	273.06	301.44	10.39%

**Table 4:** Damage Simulation and Prediction Results.

	Damage Threshold	Actual Corrosion Area (unit is mm^2^)	Predicted Corrosion Area (unit is mm^2^)	Percentage Error
Hole B	0.15	273.06	329.31	20.60%
Hole C	0.15	273.06	357.44	25.75%
